# Associated and predictive factors of depressive symptoms in patients with Parkinson’s disease

**DOI:** 10.1007/s00415-016-8130-3

**Published:** 2016-04-28

**Authors:** Kangdi Zhu, Jacobus J. van Hilten, Johan Marinus

**Affiliations:** Department of Neurology (K5Q-92), Leiden University Medical Center, P.O. Box 9600, 2300 RC Leiden, The Netherlands

**Keywords:** Depression, Parkinson’s disease, Risk factors, Prediction

## Abstract

Depression is one of the most common non-motor symptoms in Parkinson’s disease (PD). A thorough understanding of factors associated with depressive symptomatology may facilitate early detection and guide future intervention strategies. The objective of the study was to determine associated and predictive factors of depression in patients with PD. Analyses were performed in data of the SCOPA-PROPARK cohort, a 5-year hospital-based longitudinal cohort of over 400 PD patients who have been examined annually. Linear mixed models using data of all patients were used to identify factors associated with longitudinal changes in Beck Depression Inventory (BDI) scores. A survival analysis using data of patients without depression at baseline was performed to identify risk factors for future depression (i.e. BDI ≥ 15). The proportion of patients with depression was approximately 20 % and remained stable during follow-up, with approximately half of cases showing a persistent course. Female gender, more severe disability, more severe motor fluctuations, autonomic and cognitive dysfunction, poorer nighttime sleep and daytime sleepiness were independently associated with higher BDI scores over time. Higher baseline BDI score, daytime sleepiness and a higher levodopa dosage were risk factors for future depression. Depression is common in PD, where it may follow a persistent or non-persistent course. Apart from motor fluctuations and levodopa dose, depressive symptoms in PD are mainly associated with factors of non-dopaminergic origin. This suggests that depression in PD is an inherent consequence of the progressive pathobiology of the disease, which may render its treatment with currently available treatment options difficult.

**Electronic supplementary material:**

The online version of this article (doi:10.1007/s00415-016-8130-3) contains supplementary material, which is available to authorized users.

## Introduction

With a prevalence of about 40 %, depression is one of the most common non-motor symptoms of Parkinson’s disease (PD) [1]. It contributes significantly to the disease burden [2] and several studies identified depression as the main determinant of poor quality of life in PD patients [3]. Symptoms that contribute to the clinical semiology of depression show an overlap with those primarily related to PD or those related to the side effects associated with the use of medication [4]. This renders the identification of depression in PD difficult and it is assumed that this condition frequently remains unrecognized [5]. Increased knowledge of associated and risk factors of depression in PD may therefore facilitate its early detection, provide insight into the nature of this condition, and guide future intervention strategies [5, 6].

In earlier studies in PD, consistent relations have been found between depression and age, anxiety, insomnia and dementia. However, contradictory findings have been reported for the relation between depression and gender, disease stage, levodopa treatment and motor subtype [postural instability/gait difficulty (PIGD)] [7–20].

These inconsistencies are likely explained by differences between studies concerning sample size, population characteristics and study design. Most previous studies on depression in PD had a cross-sectional design and, to our knowledge, only three longitudinal studies have been performed to date [7, 10, 11]. One longitudinal, hospital-based study (*n* = 685) showed that longer disease duration, greater disability, and a positive family history of motor neuron disease were risk factors associated with the development of depression [10]. Another hospital-based study (*n* = 184) found that the severity of depression in PD varied over time, with groups showing a remittent (35 %), stable (34 %) or progressive (31 %) form [7]. The largest longitudinal, population-based case–control study performed by Becker et al. (3637 PD patients and controls) showed an almost twofold increased risk to develop depression in the patients with PD. Female gender and long-term levodopa usage emerged as the most important risk factors of depression [11]. Unfortunately, in all longitudinal studies the number of baseline features used in the analysis was limited. This specifically pertains to non-dopaminergic features, which are less sensitive to dopaminergic medication and may provide a more complete and accurate evaluation of disease severity and progression in PD [21].

The PROPARK cohort study includes over 400 PD patients who have been examined annually and followed for 5 years (i.e., six assessments) on a broad range of motor and non-motor features [22]. This cohort is therefore very well-suited to investigate which factors are associated with: (1) the presence of depression in PD; (2) the longitudinal changes in severity of depressive symptoms; and (3) the development of future depression in PD.

## Methods

### Study design and participants

Patients were recruited from neurology clinics of university and regional hospitals in the western part of The Netherlands and all fulfilled the United Kingdom Parkinson’s disease Society Brain Bank criteria for idiopathic PD [23]. The majority of patients were evaluated at the Leiden University Medical Center, but more severely affected patients were offered the possibility to be examined at their homes to prevent selective dropout. In view of the fact that we aimed to obtain information on the full spectrum of the disease, a recruitment strategy based on age at onset (< or ≥50 years) and disease duration (< or ≥10 years) was applied. We intended to recruit at least 100 patients in each of the four strata [22]. The medical ethical committee of the Leiden University Medical Center approved the PROPARK study and written informed consent was obtained from all patients [22].

### Assessment of baseline variables

At baseline (2003–2005) and the five subsequent annual visits all patients received standardized assessments. The assessments included an evaluation of demographic and clinical characteristics, family history of PD, and registration of antiparkinsonian medication. A levodopa dose equivalent (LDE) of daily levodopa and dopamine agonists dose was calculated for each patient at baseline. The total LDE is the sum of levodopa dosage equivalent (LDE-Dopa) and the dopamine agonist dosage equivalent (LDE-DA) [24]. Diagnosis and Hoehn & Yahr (H&Y) stages of the patients were ascertained at every assessment [25]. The following instruments were administered by qualified examiners: the SPES/SCOPA [26] (including sections on motor examination, activities of daily living and motor complications), the SCOPA-COG cognitive function [27], and the SCOPA-PC (psychotic symptoms; items 1–5) [28]. Over the years, there were in total five examiners, who all regularly attended retraining and recalibration sessions to prevent inter-rater variability. All patients who used dopaminergic medication were assessed during “on’’. Patients completed the following instruments themselves: the SCOPA-AUT (three autonomic domains: gastrointestinal, urinary tract and cardiovascular) [29], the SCOPA-SLEEP [with sections on nighttime sleep problems (NS) and daytime sleepiness (DS)] [30], and the Beck Depression Inventory (BDI) [31].

For all instruments except the SCOPA-COG, higher scores reflect poorer functioning. Patients were classified according to motor subtype using a ratio of tremor score (SPES/SCOPA) [26] over PIGD score (SPES/SCOPA) [27]. A total tremor or PIGD score of 0 was replaced by 0.5. Patients with a ratio value <1.0 were classified as PIGD dominant, whereas those with values from ≥1.0 were classified as non-PIGD dominant [32].

### Ascertainment of depression

Depression was assessed using the Beck Depression Inventory (BDI) [31], a valid and reliable instrument that includes 21 items with four response options (0–3). In accordance with the results of an earlier study [33], a PD patient was classified as depressed if a BDI score of 15 or higher was attained.

### Statistical analysis

Given objective 1 we first evaluated which features were associated with the presence of depression in the baseline data of our population. Cross-sectional analyses were performed to assess differences at baseline between patients with and without depression. Chi square tests were used for comparing categorical variables, while independent *t*-tests were used for comparing normally distributed continuous variables; the Mann–Whitney *U* test was used if continuous variables were not normally distributed.

For objective 2 a linear mixed models (LMM) analysis was performed using the data of all patients included in the follow-up. This method allows for the identification of baseline variables that are associated with variation in BDI scores over time. LMM take into account that repeated measures in the same subject are not independent but correlated. An advantage of this method is that it can deal with missing data in the outcome, and therefore this analysis does not have to be restricted to patients with a complete follow-up. A restricted maximum likelihood (REML) model with an autoregressive (heterogeneous) covariance structure type was used in all LMM analyses; this assumes that measurements that are closer in time are more strongly correlated than those that are further apart. Since heterogeneity between patients was expected in baseline levels and in change over time, random intercepts and random slopes were used. Baseline variables that have been found associated with depression in earlier studies were considered in the LMM. These included: age, gender, sumscore of motor impairment and activities of daily living (SPES/SCOPA), motor phenotype, presence of hallucinations (score ≥1 on item 1 of the SCOPA-PC), autonomic dysfunction score (gastrointestinal, urinary tract and cardiovascular domains), sumscore for nighttime sleep problems, sumscore of cognitive dysfunction (SCOPA-COG), dosage of antiparkinsonian medication (LDE-Dopa, LDE-DA) and the use of antidepressants.

The Hoehn and Yahr stage was not included because it is partly determined by motor phenotype and the sumscore of motor impairment and disease duration was excluded because it is partly determined by age. Anxiety scores were not taken into account in the analyses because of the strong and intricate relation with depression [34]; its inclusion could therefore have obscured the relation with other characteristics.

A few other baseline variables were added because a relation with development of depression could be presumed. These included: sumscore for daytime sleepiness, sumscore of dyskinesias and the sumscore of motor fluctuations. The relationship between variables that are associated with variation in BDI scores over time was first analyzed including only one variable at a time (unadjusted model). Additionally, an adjusted model was performed that considers the main effects of all significant baseline variables from the unadjusted model. The final model only includes the variables that were significant from the adjusted model.

For objective 3 we performed a survival analysis in the data of patients who had no depression at baseline with the same variables that were considered in the LMM, while also the baseline BDI score was added in this analysis. Survival time was calculated as the difference in years between the dates on which depression was first reported and the date of the patient’s baseline assessment. Patients were considered to have an event (‘uncensored’) if they scored ≥15 on the BDI. If a patient did not have an event during the complete follow-up, he or she was ‘withdrawn alive’ and classified as ‘censored’. In case a patient had missed 1 year and had no depression in the previous and following year, we assumed that the patient had not developed depression in that year. For the survival analysis, we first performed univariate analyses to evaluate which baseline variables were associated with future development of depression (unadjusted model). An adjusted model was performed to take the potential influence of confounders into account. The final model only includes the variables that were significant from the adjusted model and were simultaneously entered in a multivariate Cox proportional hazards’ model.

Given the potential influence of antidepressant use of on depression status, a secondary analysis was performed in which patients were classified as depressed (i.e. had an ‘event’) if they attained a score ≥15 on the BDI or used antidepressants.

Risk factors for the development of depression were calculated as hazard ratios (HR) with 95 % confidence intervals (CI), with a HR >1 indicating that the particular baseline variable is associated with a higher risk of developing depression.

Analyses were performed with the Statistical Package for the Social Sciences (SPSS) version 21.0.

## Results

**Fig. 1:**
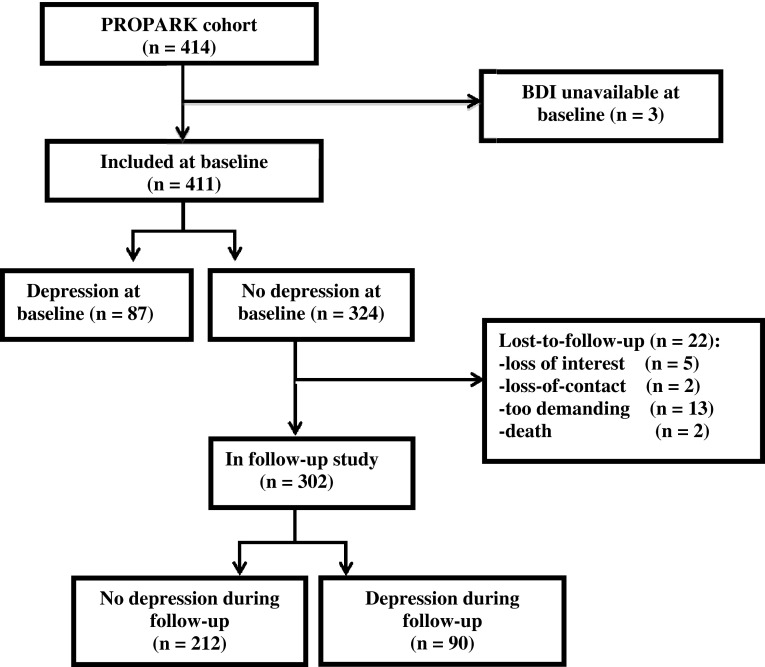
Flowchart of follow-up for depression

**Fig. 2:**
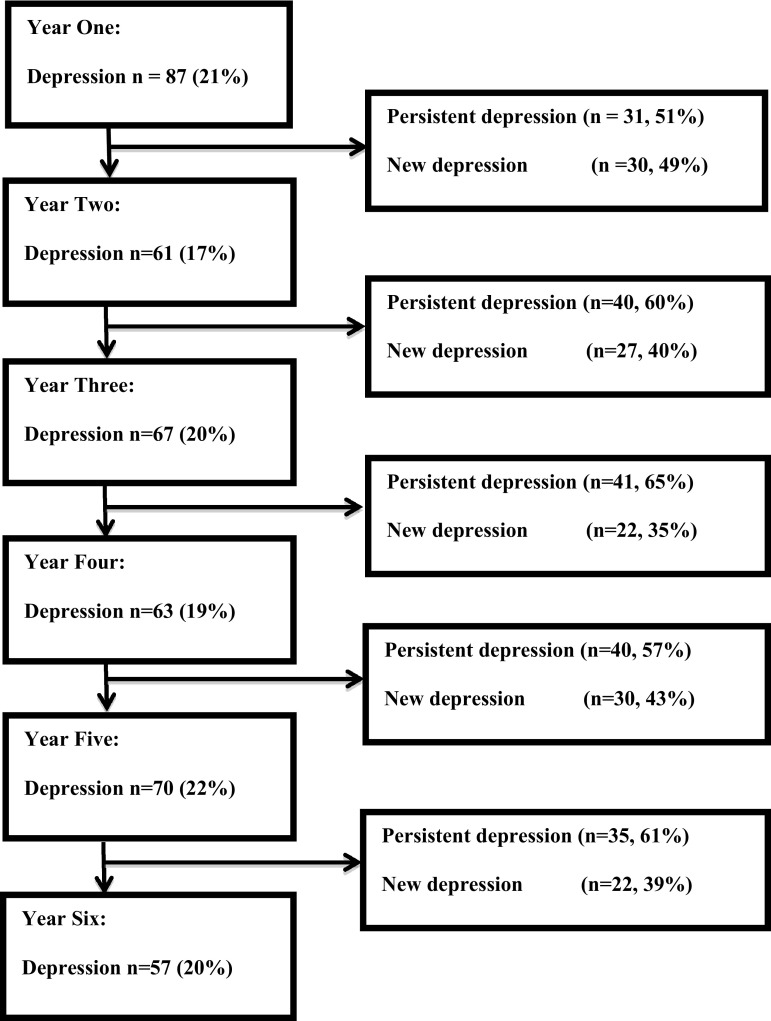
Flowchart of entire baseline population for the occurrence and persistence of depression. Percentages of persistent depression for a particular year were calculated by dividing the number of patients with persistent depression by the total number of depressed patients in the subsequent year. For instance, a total number of 61 patents were classified as depressed in year 2, of which 31 also had been classified as depressed in the previous year, resulting in a percentage of 51 (i.e., 31/61)

Of the 411 patients of whom a baseline BDI score was available, 87 (21 %) were classified as depressed and 324 patients were classified as non-depressed (see for details Fig. 1). Of the 324 patients who did not have depression at baseline, 90 patients (28 %) developed this symptom during the follow-up period. The proportion of patients with depression remained relatively stable during follow-up (from 21 % at baseline to 20 % in year 5). During the 5-year follow-up period the presence of depression among patients varied considerably, with approximately half of cases showing a persistent course (Fig. 2).

### Variables associated with depression at baseline (cross-sectional analysis)

Patients with depression at baseline were older, had a longer disease duration and higher Hoehn and Yahr stage, and performed worse with respect to motor function, activities of daily living, motor fluctuations and dyskinesias (Table 1). A significant higher proportion of patients with depression had a PIGD phenotype. They also had significantly more cognitive impairment, daytime sleepiness, nighttime sleep problems and autonomic dysfunction, and more often suffered from hallucinations. No significant differences were found regarding the use of antidepressive or antiparkinsonian medication for depressed patients as compared to non-depressed patients.

**Table 1:** Baseline data of patients with and without depression

	Total	With depression	Without depression	*p*
*N*	411	87	324	
Age (year)	61.07 (11.38)	63.65 (12.49)	60.38 (10.97)	0.02^f^
Sex (% female)	35.5	42.5	33.6	0.12^a^
Antidepressants (%)	15.4	19.5	14.2	0.22
Education (year)	11.97 (4.11)	11.47 (4.49)	12.10 (4.00)	0.20
Disease duration (year)	10.64 (6.55)	12.00 (6.67)	10.28 (6.47)	0.03^f^
Age at onset (year)	50.43 (11.87)	51.66 (11.98)	50.11 (11.84)	0.28
Hoehn and Yahr, stage	2 (2, 3)	3 (2, 4)	2 (2, 3)	<0.001^b,f^
SPES/SCOPA motor impairments	13.31 (4.90)	15.48 (5.30)	12.71 (4.59)	<0.001^f^
SPES/SCOPA Dyskinesias	0.94 (1.62)	1.41 (1.82)	0.81 (1.54)	0.006^f^
SPES/SCOPA motor fluctuations	0.78 (1.26)	1.19 (1.57)	0.67 (1.14)	0.006^f^
SPES/SCOPA ADL	8.92 (3.56)	10.86 (3.93)	8.40 (3.28)	<0.001^f^
Motor phenotype, PIGD dominant (%)	45.1	71.1	38.2	<0.001^a,f^
Beck depression inventory	10.21 (6.57)	20.06 (5.86)	7.57 (3.54)	<0.001^f^
SCOPA-COG^c^	25.32 (6.67)	22.13 (7.54)	26.18 (6.15)	<0.001^f^
SCOPA-SLEEP, NS^d^	4.52 (3.77)	7.12 (3.87)	3.83 (3.44)	<0.001^f^
SCOPA-SLEEP, EDS^d^	4.88 (3.74)	6.14 (3.83)	4.54 (3.64)	<0.001^f^
SCOPA-AUT, GI score^e^	2.72 (2.20)	3.79 (2.31)	2.43 (2.08)	<0.001^f^
SCOPA-AUT, UR score^e^	6.72 (4.03)	8.46 (4.46)	6.28 (3.79)	<0.001^f^
SCOPA-AUT, CV score^e^	1.16 (1.19)	1.83 (1.37)	0.98 (1.08)	<0.001^f^
Hallucinations, % with	17.0	30.0	13.7	0.001^a,f^
Total LDE (mg/day)	609 (464)	670 (423)	593 (474)	0.17
LDE-Dopa (mg/day)	380 (375)	441 (363)	363 (378)	0.09
LDE-DA dose (mg/day)	232 (226)	229 (218)	232 (229)	0.90

Variables are expressed as means (standard deviations), except for gender (percentages), motor subtype (percentages) and Hoehn and Yahr stage [median ((interquartile range)]. All differences are calculated with the independent-sample *t* tests, except for ^a^ Chi square test and ^b^ Mann–Whitney *U* test

*DBS* deep brain surgery, *ADL* activities of daily living, *PIGD* postural instability/gait difficulty, *BDI* Beck depression inventory, *LDE* Levodopa dosage equivalent, *DA* dopamine agonists

^c^SCOPA-COG: cognitive function, higher scores reflect better functioning

^d^SCOPA-SLEEP, NS score: nighttime sleep problems; DS score: daytime sleepiness

^e^SCOPA-AUT: sumscore autonomic functioning including items from the sections on gastrointestinal (GI), cardiovascular (CV) and urinary tract (UR)

^f^Significant values

### Variables associated with longitudinal changes in BDI (LMM analysis)

The final model of the LMM analysis showed that female gender, more difficulties with activities of daily living and motor fluctuations, more cognitive impairment, more nighttime sleep problems and increased daytime sleepiness at baseline were associated with higher BDI scores over time (Table 2). In addition, autonomic dysfunction (urinary and cardiovascular domains) and the use of antidepressive medication were significantly related to higher BDI scores.

**Table 2:** Factors associated with higher BDI scores over time in patients with PD

Variable	Unadjusted model^a^		Adjusted model^b^		Final model^c^	
	*B* (95 % CI)	*p*	*B* (95 % CI)	*p*	*B* (95 % CI)	*p*
Age	0.10 (0.07–0.12)	<0.001^g^	−0.01 (−0.03 to 0.03)	0.85		
Female gender	1.62 (1.04–2.21)	<0.001^g^	1.08 (0.49 to 1.67)	<0.001^g^	0.96 (0.44–1.48)	<0.001^g^
SPES/SCOPA—motor impairment	0.33 (0.26–0.39)	<0.001^g^	0.05 (−0.03 to 0.12)	0.25		
SPES/SCOPA—ADL	0.63 (0.55–0.70)	<0.001^g^	0.14 (0.01–0.26)	0.04^g^	0.16 (0.07–0.25)	<0.001^g^
SPES/SCOPA—Dyskinesia	0.67 (0.50–0.85)	<0.001^g^	−0.12 (−0.33 to 0.09)	0.25		
SPES/SCOPA—motor fluctuations	1.20 (0.97–1.42)	<0.001^g^	0.30 (0.06–0.54)	0.02^g^	0.35 (0.14–0.56)	0.001^g^
PIGD dominant phenotype	2.71 (2.14–3.28)	<0.001^g^	0.13 (−0.48 to 0.74)	0.68		
SCOPA-COG score^d^	−0.30 (−0.34 to 0.26)	<0.001^g^	−0.20 (−0.25 to 0.15)	<0.001^g^	−0.19 (−0.23 to 0.14)	<0.001^g^
Presence of hallucinations	3.60 (2.83–4.36)	<0.001^g^	0.25 (−0.55 to 1.05)	0.54		
SCOPA-SLEEP-NS score^e^	0.53 (0.46–0.60)	<0.001^g^	0.43 (0.35–0.50)	<0.001^g^	0.47 (0.40–0.54)	<0.001^g^
SCOPA-SLEEP-DS score^e^	0.51 (0.37–0.66)	<0.001^g^	0.23 (0.15–0.31)	<0.001^g^	0.25 (0.18–0.32)	<0.001^g^
SCOPA-AUT^f^ GI score	0.85 (0.73–0.98)	<0.001^g^	0.28 (0.14–0.43)	<0.001^g^	0.10 (−0.03 to 0.23)	0.13
SCOPA-AUT^f^ CV score	1.61 (1.37–1.84)	<0.001^g^	0.45 (0.19–0.72)	0.001^g^	0.36 (0.13–0.60)	0.002^g^
SCOPA-AUT^f^ UR score	0.57 (0.50–0.64)	<0.001^g^	0.13 (0.04–0.21)	0.003^g^	0.18 (0.11–0.25)	<0.001^g^
Daily levodopa dose, *p*/100 mg	0.40 (0.32–0.48)	<0.001^g^	−0.04 (−0.13 to 0.06)	0.44		
Daily DA dose, *p*/100 mg	0.12 (−0.14 to 0.37)	0.37				
Use of antidepressants	2.82 (2.01–3.62)	<0.001^g^	1.52 (0.75–2.30)	<0.001^g^	1.55 (0.86–2.24)	<0.001^g^

Estimates are presented as B with 95 % confidence intervals (CI), where a positive value is associated with a positive relationship between the baseline variable and BDI scores

*ADL* activities of daily living,* PIGD* postural instability/gait difficulty,* BDI* Beck depression inventory,* DA* dopamine agonists

^a^The unadjusted model between BDI scores and the baseline variables were analyzed including one covariate at a time

^b^The adjusted model includes only the significant variables (*p* < 0.05) from the unadjusted model

^c^The final model includes only the significant variables (*p* < 0.05) from the adjusted model

^d^SCOPA-COG: cognitive function, higher scores reflect better functioning

^e^SCOPA-SLEEP, DS: daytime sleepiness NS: Nighttime sleep problems

^f^SCOPA-AUT: sumscore autonomic functioning including items from the sections on gastrointestinal (GI), cardiovascular (CV) and urinary tract (UR)

^g^Significant values

### Variables associated with persistent depression

Of the total of 354 patients of whom at least three measurements were available, 152 were classified as depressed either at baseline or during one of the follow-up assessments (Fig. 2). Of these 152 patients, 58 patients had a persistent form of depression (i.e. >50 % of assessments qualifying for depression) and 94 patients had a non-persistent form (≤50 % of assessments qualifying for depression).

For patients with a persistent form of depression, the median (interquartile range) number of episodes of depression was 4 (3, 5), whereas for patients with a non-persistent form the median was 1 (1, 2). In comparison with baseline values of patients with non-persistent depression, patients with persistent depression were older, more often female, longer diseased, and also had more severe motor impairments (SPES-Motor and H&Y) and cognitive impairment (Supplemental Table 1). In addition, at baseline these patients already exhibited more severe depressive symptoms and were more often treated with antidepressants.

### Risk factors for future development of depression (survival analysis)

The multivariate Cox proportional hazards’ model showed that a higher baseline BDI score, daytime sleepiness and a higher levodopa dosage were independent predictors for future development of depression in patients who were non-depressed at baseline (Table 3).

**Table 3:** Longitudinal risk factor analysis of the development of depression in patients without depression at baseline

	Unadjusted model^a^		Adjusted model^b^		Final model^c^	
	HR (95 % CI)	*p*	HR (95 % CI)	*p*	HR(95 % CI)	*p*
Age, p/year increase	1.03 (1.01–1.05)	0.007^g^	1.01 (0.98–1.04)	0.45		
Gender, HR for females	1.09 (0.71–1.67)	0.70				
Baseline BDI score, p/point increase	1.31 (1.23–1.40)	<0.001^g^	1.29 (1.19–1.40)	<0.001^g^	1.27 (1.18–1.36)	<0.001^g^
Disease duration, p/year increase	1.01 (0.98–1.05)	0.38				
SPES/SCOPA—motor impairments	1.04 (0.98–1.09)	0.18				
SPES/SCOPA—ADL	1.11 (1.04–1.18)	0.001^g^	0.98 (0.91–1.06)	0.64		
SPES/SCOPA—Dyskinesia	1.12 (0.99–1.27)	0.07				
SPES/SCOPA—motor fluctuations	1.24 (1.06–1.46)	0.008^g^	0.92 (0.75–1.13)	0.42		
Motor phenotype, HR for PIGD dominant	1.56 (1.02–2.38)	0.04^g^	0.90 (0.54–1.50)	0.69		
SCOPA-COG^d^, p/point increase	0.95 (0.92–0.98)	0.002^g^	0.97 (0.93–1.01)	0.18		
Presence of hallucinations, yes/no	2.11 (1.23-3.64)	0.007^g^	1.42 (0.78–2.59)	0.26		
SCOPA-SLEEP-DS^e^, p/point increase	1.16 (1.10–1.22)	<0.001^g^	1.11 (1.05–1.18)	0.001^g^	1.10 (1.04–1.17)	0.001^g^
SCOPA-SLEEP-NS^e^, p/point increase	1.09 (1.03–1.15)	0.002^g^	0.99 (0.92–1.06)	0.68		
SCOPA-AUT, GI^f^ score p/point increase	1.01 (1.00–1.21)	0.05^g^	0.92 (0.82–1.03)	0.16		
SCOPA-AUT, CV^f^ score p/point increase	1.33 (1.13–1.34)	0.001^g^	1.10 (0.89–1.35)	0.38		
SCOPA-AUT, UR^f^ score p/point increase	1.09 (1.03–1.14)	0.002^g^	0.98 (0.91–1.05)	0.60		
Daily levodopa dose, p/100 mg increase	1.12 (1.07–1.18)	<0.001^g^	1.12 (1.03–1.21)	0.006^g^	1.09 (1.03–1.15)	0.004^g^
Daily DA dose, p/100 mg increase	1.12 (1.03–1.21)	0.007^g^	1.08 (0.97–1.20)	0.15		
Use of antidepressants, yes/no	1.51 (0.87–2.63)	0.15				

All variables are expressed as hazard ratio (HR) with 95 % confidence interval (CI)

*ADL* activities of daily living, *PIGD* postural instability/gait difficulty, *BDI* Beck depression inventory, *DA* dopamine agonists

^a^The unadjusted model between BDI scores and the baseline variables were analyzed including one covariate at a time

^b^The adjusted model includes only the significant variables (*p* < 0.05) from the unadjusted model

^c^The final model includes only the significant variables (*p* < 0.05) from the adjusted model

^d^SCOPA-COG: cognitive function, higher scores reflect better functioning

^e^SCOPA-SLEEP, DS score: daytime sleepiness NS: Nighttime sleep problems

^f^SCOPA-AUT: sumscore autonomic functioning including items from the sections on gastrointestinal (GI), cardiovascular (CV) and urinary tract (UR)

^g^Significant values

For the secondary analysis, also patients using antidepressive medication were classified as depressed, which resulted in an increase of patients classified as depressed at baseline and an inherent decrease of the population at risk for future development of depression. In this scenario 89 patients out of a total of 272 developed depression during follow-up; 21 of those 89 patients were classified as depressed solely because of antidepressant use. The same three variables (higher baseline BDI score, increased daytime sleepiness and a higher levodopa dosage) emerged from the multivariate Cox proportional hazards’ model.

## Discussion

Depression in PD likely results from complex interactions among genetic vulnerabilities, cognitive predisposition, age-associated neurobiological changes and stressful events. Although deficiencies in the dopaminergic, serotonergic and cholinergic networks have all been suggested to play a role in the pathobiology of depression in PD [35, 36], the multisystem nature of the disease renders it difficult to pinpoint the specific causes of depression in this condition. Against this background, knowledge of associated and risk factors of depression may provide insight into the nature of depression in PD.

In this study, we examined the presence and course of depression over 5 years in a large cohort of over 400 patients with PD. The prevalence of depression during follow-up was stable, at approximately 20 %, which corresponds with findings of another longitudinal hospital-based study [10]. We further found that depression may persist or show a non-persistent course, which corroborates with findings of the study by Rojo et al. [7].Compared to patients with a non-persistent course, patients with persistent depression were older, more often female and longer diseased. Interestingly, these patients had more severe depressive symptomatology at baseline, even though they were more often treated with antidepressants. Our findings further suggest that patients with persistent depression suffer more advanced PD.

One might wonder if PD patients with persistent depression (*n* = 58) differed in progression on other non-motor and motor domains as compared to patients who were persistently non-depressed (*n* = 202). After performing an additional analysis in which we adjusted for differences in age, gender and disease duration, we found that persistent depression was associated with worse performance over time on all domains. (Supplemental Table 2).

### “Which factors are associated with longitudinal changes in depressive symptoms?”

The analysis of baseline differences between depressed and non-depressed PD patients provided information on the variables that potentially should be taken into account in the longitudinal analysis. In the longitudinal analysis we found that female gender, more severe disability, more cognitive impairment, motor fluctuations, nighttime sleep problems, increased daytime sleepiness, more autonomic dysfunction (urinary and cardiovascular domains) and the use of antidepressants were independently associated with higher BDI scores over time (LMM).

Studies evaluating depression in PD have usually examined a limited number of clinical variables and the results among these studies were often inconclusive due to heterogeneity of sample compositions and the cross-sectional nature of the study designs. As a result, contradictory findings have been reported.

Female gender, more severe disability and lower cognition scores were variables found to be associated with more severe depressive symptoms, which is in agreement with results from two earlier longitudinal studies [7, 10]. We further found that motor fluctuations, nighttime sleep problems and autonomic dysfunction were associated with depressive symptomatology, findings that only have been found in previous cross-sectional studies (Supplemental Table 3). We identified one other associated factor of depression, namely daytime sleepiness. Interestingly, this symptom, together with depression, cognitive decline, autonomic dysfunction, psychotic symptoms and PIGD were previously identified as components of a coherent predominantly non-dopaminergic (PND) symptom complex in PD [37]. Notably, this complex is prevalent early in the disease and worsens with disease progression [21], which likely is the consequence of progressive α-synuclein aggregate-related synaptopathy and axon degeneration of the nervous system [38–40]. All five other components of the PND complex were associated with higher BDI scores over time, of which three made an independent contribution to the model (daytime sleepiness, cognitive impairment and autonomic dysfunction). Interestingly, compared to patients not on antidepressants, patients on antidepressants had higher BDI scores and suffered more advanced PD. [mean (SD) BDI 12.38 (7.02) vs 9.83 (6.42); *p* = .004]. Collectively, these findings suggest that progression of pathobiology is an important causative factor for depression in PD, which might be resistant to currently available treatment options for depression.

Motor fluctuations were also found to be associated with depressive symptoms and this complication of levodopa treatment usually increases in prevalence and severity as the progression of PD advances. In non-depressed PD patients motor fluctuations may be associated with mood fluctuations [8]. Since several effective strategies to target motor fluctuations are now available [41], these approaches potentially may also have an impact on depressive symptoms in PD.

### “Which factors are associated with an increased risk of future depression?”

Approximately 28 % of patients who had no depression at baseline fulfilled the criteria for depression at least once during the course of the study (Fig. 1). The presence of depression across these patients varied considerably each year, with approximately half of the cases showing persistent depression while the other half showed depression with a non-persistent pattern. Because of the potential overlap in somatic symptoms of depression and PD, we also examined if at least one or both of the two non-somatic symptoms that are essential for the clinical diagnosis of major depression [42], i.e., feeling sad (item 1 of the BDI) and loss of pleasure (item 4), were present in those classified as depressed. This analysis showed that at least one of these features was present in 97 % of patients who were classified as depressed (BDI >15) at baseline, and in 93 % of patients who were classified as depressed during follow-up. This indicates that non-somatic features were included the classification of depression in the vast majority of cases.

The survival analysis showed that higher baseline BDI scores, increased daytime sleepiness and higher levodopa dosage were risk factors for future depression. As mentioned earlier, a higher baseline BDI score was also an important predictor for a persisting form of depression. Similar to the findings by Becker et al., levodopa dose emerged as an independent risk factor for future depression in our study [10]. Interestingly, levodopa only emerged in the survival analysis and not the LMM. To date, however, the role of levodopa in depression of PD has remained controversial, with studies reporting effects varying from protection to deterioration [43, 44]. Serotonin is a key factor in mood regulation and in a rat model long-term levodopa treatment decreased serotonin synthesis in the nucleus raphe dorsalis and other serotonergic regions in the brain [45]. We can therefore not exclude that over time, continued exposure to levodopa contributes to the development of depression in PD. The finding that daytime sleepiness is a predictor of future development of depression corresponds with our findings from the LMM analysis.

Of note is that 4–17 % of all patients who were depressed were treated with antidepressants over the years of the study. Since no information was available on the efficacy of drugs used to treat depression in our cohort, the use of antidepressants was not considered in the classification of patients in the primary analysis of this study, although we controlled for use of this medication by including it as a covariate. In a secondary analysis patients who had a BDI < 15 but used antidepressants were also classified as depressed and this approach revealed similar results, supporting the robustness of the findings.

Of note is that the dopamine agonist pramipexole has been found to have antidepressant properties in a randomized clinical trial setting [44]. In our cohort, 26 % of patients used this medication at baseline and this could have impacted the occurrence and course of depressive symptoms. We therefore performed an additional univariate LMM analysis where use of pramipexole (yes/no) was included as a separate variable and this analysis showed that this variable was not significantly associated with BDI scores over time [*B*(95 % CI) = −0.18(−1.43 to 1.07), *p* = 0.78], which makes potential confounding by use of this dopamine agonist unlikely. The application of a cutoff score to classify patients as depressed or not depressed and the non-persistent course of depression could have contributed to the apparent discrepancy between the results of the LMM and the Cox Proportional Hazards model. Although both procedures involve analysis of longitudinal data, they provide different answers to different questions, namely: “Which factors are associated with longitudinal changes in depressive symptoms?” (LMM) vs “Which factors are associated with an increased risk of future depression in patients who are free of this condition at baseline?” (survival analysis). In addition, data of all patients are used in the LMM analysis, whereas in the survival analysis only data of patients who are free of depression at baseline are used.

The strengths of this study are the prospective design, the broad clinical characterization, the limited loss to follow-up and the size of the cohort. Limitations of our study relate to the fact that we were not knowledgeable of previously reported patient-specific baseline risk factors of depression, namely the occurrence of life events, personality traits, history of depression, pain or fatigue [12, 17]. In addition, due to an overlap of symptoms of depression and PD, one could argue that it is not surprising that the severity of PD, or a higher baseline BDI score, would predict future BDI scores. However, we attempted to control this potentially distorting effect on our results using a PD-specific cutoff value for depression of the BDI and by applying a multivariate approach, where, amongst others, differences in baseline disease severity and duration were taken into account. At last, our cohort is hospital-based, which may have resulted in some over- or underestimation, although it seems unlikely that this has resulted in significant distortions of our conclusions.

In summary, in this prospectively studied cohort of patients with PD, depression is a common feature that may follow a persistent or a non-persistent course and occurs more often in female patients. Apart from motor fluctuations and levodopa dose, depressive symptoms in PD are mainly associated with factors of non-dopaminergic origin. This suggests that depression in PD is an inherent consequence of the progressive pathobiology of the disease, which may render its treatment with currently available treatment options difficult.

## Electronic supplementary material

Below is the link to the electronic supplementary material.

Supplementary material 1 (DOCX 42 kb)
