# Supplementary material for: Associated and predictive factors of depressive symptoms in patients with Parkinson’s disease
Source: J Neurol. 2016 Apr 28;263:1215–25. doi: 10.1007/s00415-016-8130-3 (PMC4893359; doi:10.1007/s00415-016-8130-3)
Supplement: Supplementary file 1 — Supplementary material 1 (DOCX 42 kb) [file 415_2016_8130_MOESM1_ESM.docx]

**Supplemental Table 1 - Baseline data of patients with and without persisting depression**

|  | **Total** | **Persisting depression** | **Non-persisting depression** | **P** |
| --- | --- | --- | --- | --- |
|  |  |  |  |  |
| N | 152 | 58 | 94 |  |
| Age, yr | 61.73 (10.72) | 63.90 (11.11) | 60.39 (10.30) | .05^f^ |
| Sex, % female | 40.1 | 50.0 | 34.0 | .05^a,f^ |
| Antidepressants, % | 19.0 | 31.0 | 11.7 | .003^a,f^ |
| Education, yr | 11.77 (4.09) | 11.11 (4.15) | 12.18 (4.02) | .12 |
| Disease duration, yr | 11.12 (5.77) | 12.42 (6.16) | 10.31 (5.40) | .03^f^ |
| Age at onset, yr | 50.62 (10.38) | 51.49 (10.72) | 50.08 (10.19) | .42 |
| Hoehn & Yahr, stage | 3 (2,3) | 3 (2,4) | 2 (2,3) | .001^b,f^ |
| SPES/SCOPA  Motor Impairments | 13.88 (5.06) | 15.11 (5.40) | 13.12 (4.71) | .02^f^ |
| SPES/SCOPA  Dyskinesias | 1.16 (1.71) | 1.35 (1.78) | 1.04 (1.66) | .29 |
| SPES/SCOPA  Motor Fluctuations | 1.04 (1.39) | 1.04 (1.29) | 1.04 (1.46) | .98 |
| SPES/SCOPA ADL | 9.59 (3.34) | 10.25 (3.59) | 9.18 (3.12) | .06 |
| Motor phenotype,  PIGD dominant, % | 51.9 | 62.0 | 45.9 | .08^a^ |
| Beck Depression Inventory | 14.22 (6.70) | 18.07 (6.77) | 11.85 (5.47) | <.001^f^ |
| SCOPA-COG^c^ | 24.91 (5.67) | 22.93 (5.90) | 26.09 (5.22) | .001^f^ |
| SCOPA-SLEEP, NS^d^ | 6.09 (3.86) | 6.50 (4.09) | 5.83 (3.72) | .30 |
| SCOPA-SLEEP, EDS^d^ | 6.10 (3.95) | 6.71 (4.00) | 5.72 (3.90) | .14 |
| SCOPA-AUT, GI score^e^ | 3.12 (3.19) | 3.36 (2.18) | 2.97 (2.19) | .28 |
| SCOPA-AUT, UR score^e^ | 7.68 (4.01) | 8.40 (4.10) | 7.24 (3.91) | .08 |
| SCOPA-AUT, CV score^e^ | 1.46 (1.30) | 1.63 (1.23) | 1.36 (1.33) | .22 |
| Hallucinations, % with | 23.1 | 26.9 | 20.9 | .41^a^ |
| LDE-Dopa, mg/day | 451 (375) | 462 (370) | 445 (379) | .79 |
| LDE-DA dose, mg/day | 276 (231) | 262 (243) | 284 (225) | .56 |

Only patients from whom at least 3 measurements were available are included in this table. Patients were considered to have a persisting form of depression if he or she was classified as depressed for more than 50% of the total number of assessments (i.e. BDI score ≥15) and a non-persisting form of depression if he or she was classified as depressed for lesser than or equal to 50% of the total number of assessments (i.e. BDI score <15).

Variables are expressed as means (standard deviations), except for gender, antidepressants, motor subtype, hallucinations (percentages) and Hoehn and Yahr stage (median ((interquartile range)). All differences are calculated with the independent-samples t-tests, except for ^a^ Chi-square test and ^b^ Mann-Whitney U test.

^c^ SCOPA-COG: cognitive function, higher scores reflect better functioning.

^d^ SCOPA-SLEEP, NS score: nighttime sleep problems; DS score: daytime sleepiness

^e^ SCOPA-AUT: sumscore autonomic functioning including items from the sections on gastrointestinal (GI), cardiovascular (CV) and urinary tract (UR).

^f^ Significant values

Abbreviations: ADL, activities of daily living; PIGD, postural instability gait difficulty; BDI, Beck depression inventory; DA, dopamine agonists.

**Supplemental Table 2 - Progression of scores on different domains for patients with persistent depression vs persistent non-depression, after adjusting for age, gender and disease duration.**

| **Variable** | **B (95%CI)** | **P** |  |
| --- | --- | --- | --- |
| SPES/SCOPA – Motor Impairment | 2.79 (1.64-3.94) | <.001^e^ |  |
| SCOPA-COG score^a^ | -3.52 (-5.00- -2.05) | <.001^e^ |  |
| SPES/SCOPA – ADL | 2.45 (1.64-3.27) | <.001^e^ |  |
| SPES/SCOPA – Motor Fluctuations | 1.01 (0.47-1.54) | <.001^e^ |  |
| SPES/SCOPA – Dyskinesia | 0.39 (0.04-0.73) | .03^e^ |  |
| PIGD score^b^ | 1.60 (1.07-2.13) | <.001^e^ |  |
| SCOPA-SLEEP-NS score^c^  SCOPA-SLEEP-DS score^c^  SCOPA-AUT^d^ GI score  SCOPA-AUT^d^ UR score  SCOPA-AUT^d^ CV score | 2.54 (1.73-3.35)  2.37 (1.45-3.29)  0.67 (0.18-1.16)  1.88 (1.01-2.76)  0.69 (0.42-0.97) | <.001^e^  <.001^e^  .007^e^  <.001^e^  <.001^e^ |  |
| SCOPA – PC^e^ – Hallucinations | 0.23 (0.11-0.35) | <.001^e^ |  |

Estimates are presented as B with 95% confidence intervals (CI), where a positive value is associated with a positive relationship between persistent depression and the specified domain.

^a^ SCOPA-COG: cognitive function, higher scores reflect better functioning.

^b^ PIGD score: sumscore of postural-instability and gait disorder (problems with freezing, gait, postural stability and walking)

^c^ SCOPA-SLEEP, DS: daytime sleepiness NS: Nighttime sleep problems

^d^ SCOPA-AUT: sumscore autonomic functioning including items from the sections on gastrointestinal (GI), cardiovascular (CV) and urinary tract (UR).

^e^ SCOPA-PC, hallucinations subscore of scale on psychiatric complications (PC)

^f^ significant values

**Supplemental Table 3 - Risk factors associated with developing depression or depressive symptoms in PD**

| **Risk Factor** | **References from studies supporting association with depression** | **References from studies not supporting an association with depression** |
| --- | --- | --- |
| Older age | 6 | 2,7,12 |
| Female gender^b^ | 1, 5^a^,9 | 2,3 |
| Disease duration | 4^a^,11 |  |
| Severity motor symptoms | 2,4^a^,7,8,11,  12 | 6,13 |
| Severity of disability (ADL)^b^ | 1 |  |
| PIGD dominant subtype | 11 | 3 |
| Motor fluctuations^b^ | 2,11 |  |
| Cognitive symptoms^b^ | 1,2,11 |  |
| Nighttime sleep problems^b^ | 10,11 |  |
| Daytime Sleepiness^b^ |  |  |
| Autonomic symptoms^b^ | 11,13 |  |
| Levodopa dosage^b^ | 2,5^a^ |  |
| Hallucinations | 11,14 |  |

^a^ Risk factor observed in longitudinal study in existing literature (all other studies had a cross-sectioonal design);

^b^ Factor associated with depressive symptoms or development of future depression confirmed by either LMM or survival analysis in the current study

List of references (in text references 7-20)

1. Rojo A, Aguilar M, Garolera MT, et al (2003) Depression in Parkinson’s disease: Clinical correlates and outcome. Parkinsonism Relat Disord 10(1):23–28.
2. Tandberg E, Larsen JP, Aarsland D, Laake K, Cummings JL (1997) Risk factors for depression in Parkinson disease. Arch Neurol 54(5):625–630.
3. van der Hoek TC, Bus BA, Matui P, van der Marck MA, Esselink RA, Tendolkar I (2011) Prevalence of depression in Parkinson's disease: effects of disease stage, motor subtype and gender. J Neurol Sci 310(1-2):220-224.
4. Jasinska-Myga B, Putzke JD, Wider C, Wszolek ZK, Uitti RJ (2010) Depression in Parkinson’s disease. Can J Neurol Sci 37(1):61-66.
5. Becker C, Brobert GP, Johansson S, Jick SS, Meier CR (2011) Risk of incident depression in patients with Parkinson disease in the UK. Eur J Neurol 18(3):448-453.
6. Leentjens AF, Lousberg R, Verhey FR (2002) Markers for depression in Parkinson’s disease. Acta Psychiatr Scand 106(3):196–201.
7. Schrag A, Jahanshahi M, Quinn NP (2001) What contributes to depression in Parkinson’s disease? Psychol Med 31(1):65–73.
8. Starkstein SE, Preziosi TJ, Bolduc PL, Robinson RG (1990) Depression in Parkinson’s disease. J Nerv Ment Dis 178(1):27–31.
9. Kuopio AM, Marttila RJ, Helenius H, et al (2000) The quality of life in Parkinson’s disease. Mov Disord 15(2):216–223.
10. Verbaan D, van Rooden SM, Visser M, Marinus J, van Hilten JJ (2008) Nighttime sleep problems and daytime sleepiness in Parkinson’s disease. Mov Disord 23(1):35–41.
11. Dissanayaka NN, Sellbach A, Silburn PA, O'Sullivan JD, Marsh R, Mellick GD (2011) Factors associated with depression in Parkinson's disease. J Affect Disord 132(1-2):82-88.
12. Kostic VS, Filipovic SR, Lecic D, et al (1994) Effect of age at onset on frequency of depression in Parkinson’s disease. J Neurol Neurosurg Psychiatry 57(10):1265–1267.
13. Berrios GE, Campbell C, Politynska BE (1995) Autonomic failure, depression and anxiety in Parkinson's disease. Br J Psychiatry 166(6):789-792.
14. Aarsland D, Larsen JP, Cummins JL, Laake K (1999) Prevalence and clinical correlates of psychotic symptoms in Parkinson disease: A community-based study. Arch Neurol 56(5):595–601.
